# Pain during exacerbation of chronic obstructive pulmonary disease: A prospective cohort study

**DOI:** 10.1371/journal.pone.0217370

**Published:** 2019-05-24

**Authors:** Maxime Maignan, Jean-Marc Chauny, Raoul Daoust, Ludivine Duc, Prudence Mabiala-Makele, Roselyne Collomb-Muret, Matthieu Roustit, Caroline Maindet, Jean-Louis Pépin, Damien Viglino

**Affiliations:** 1 Grenoble-Alps University Hospital, Emergency Department and Mobile Intensive Care Unit, Grenoble, France; 2 INSERM U1042, HP2 Laboratory, University Grenoble, Alps, Grenoble, France; 3 Department of Emergency Medicine, Research Centre, Sacré-Coeur Hospital of Montreal, Montreal, Quebec, Canada; 4 Clinical Pharmacology Department, INSERM CIC1406, University Hospital, Grenoble, France; 5 Grenoble-Alps University Hospital, Pain Medicine Department, Grenoble, France; 6 Grenoble-Alps University Hospital, Department of Physiology and Sleep, Grenoble, France; University of Notre Dame Australia, AUSTRALIA

## Abstract

**Background and objective:**

Pain, a symptom often present in patients with Chronic Obstructive Pulmonary Disease (COPD), alters quality of life. COPD exacerbation augments several mechanisms that may cause pain (dyspnea, hyperinflation and inflammation) and therefore we hypothesized that pain might be increased during exacerbation.

**Methods:**

A prospective cohort study was conducted in patients admitted for acute exacerbations of COPD (AECOPD) in two emergency departments in France and Canada. Patients with cancer-related pain or recent trauma were not included. The Short Form McGill Pain Questionnaire (SF-MPQ) and the Brief Pain Inventory (BPI) scale were used to evaluate pain intensity and location. Patients also completed the Borg Dyspnea Scale and Hospital Anxiety and Depression Scale. The questionnaires were completed again during an outpatient assessment in the stable phase. The primary outcome was difference in pain intensity (SF-MPQ) between the exacerbation and stable phases.

**Results:**

Fifty patients were included. During exacerbation, 46 patients (92%) reported pain compared to 29 (58%) in the stable phase (p<0.001). Pain intensity was higher during exacerbation (SF-MPQ 29.7 [13.6–38.2] vs. 1.4 [0.0–11.2]; p<0.001). Pain was predominantly located in the chest during exacerbation and in the limbs during the stable phase. Pain intensity during exacerbation correlated with anxiety score.

**Conclusion:**

The frequency and intensity of pain were higher during AECOPD, with a specific distribution. Pain should therefore be routinely assessed and treated in patients with AECOPD.

## Introduction

The current aim of treatment for exacerbations of chronic obstructive pulmonary disease (COPD) is to relieve the respiratory symptoms, improve respiratory function and normalize blood gases [1]. COPD is, however, a multi-dimensional disorder and the treatment of comorbidities and extra-pulmonary symptoms is emerging as equally important during acute exacerbations (AECOPD) [2]. Care-strategies now incorporate cardiovascular comorbidities, muscle dysfunction and nutritional aspects, as well as the management of anxiety and pain [2,3].

Pain is a frequently reported symptom in patients with COPD, even in non-palliative stages of the disease [4–8]. Twenty-one to 72% of patients with stable COPD report pain [7]. Pain has a major impact on quality of life as it contributes both to a reduction in physical activity and the development of mood disorders [9,10]. The underlying mechanisms of pain in COPD are not clear, although musculoskeletal disorders, comorbidities and systemic inflammation have all been proposed as contributing factors, along with increased work during breathing and increased respiratory muscle activity [6,7,11]. One study showed that dyspnea, but not lung function, was associated with pain [12] but otherwise, to date, few studies have addressed the question of whether pain increases during AECOPD.

We hypothesized that since the factors that have been associated with increased pain, as listed above, worsen during exacerbations, this could also lead to increased pain intensity and/or the number of pain-sites. A better understanding of pain during COPD exacerbations could improve its management. Effective analgesia would also facilitate patient compliance with AECOPD treatments and early rehabilitation [13,14].

The primary aim of this study therefore was to compare patient-reported COPD pain intensity during the stable phase and during exacerbation. The secondary aims were to determine if the location of pain changed between the stable and exacerbation phases and to screen for associations between pain intensity and symptoms of dyspnea, anxiety/depression, GOLD stage, exacerbation severity, and the need for noninvasive or invasive ventilation and/or the necessity for intensive care unit admission.

## Methods

### Study design and population

This prospective cohort study was conducted in two university hospitals (Grenoble, France, and Montréal, Québec, Canada). The protocol (Clinical Trial Registration NCT03102970) was approved by the ethics committees of both institutions (CECIC Rhône-Alpes, France IRB 2016–1247; Hôpital Sacré-Coeur, Montréal, Canada IRB 2015–44). All information regarding procedures and data privacy were made known to the participants before obtaining signed consent. Patients were included if they were admitted for an AECOPD while still in the emergency department. Exacerbation was defined, according to the recommendations of the Global Obstructive Lung Disease Initiative (GOLD), as an acute event characterized by a deterioration of respiratory symptoms that was greater than usual daily variations and which required an adjustment of treatment [2]. Other inclusion criteria were: having undergone at least one previous respiratory function test with a FEV1 / FVC ratio <0.7 post-bronchodilator, aged 40 years (or older) and having a tobacco consumption of at least 10 pack / years (active or former smoker). The main non-inclusion criteria were pregnancy or breastfeeding, reported pain that arose from either chronic cancer or a trauma (fracture, dislocation or severe sprain) that had occurred within 15 days prior to inclusion, or an inability to communicate (including language barriers or any documented diagnosis of dementia).

### Procedures

At inclusion, participants were asked to complete several questionnaires (see the Measurements and Outcomes section) within six hours of admission (exacerbation phase). Those with severe respiratory decompensation who were unable to communicate because of dyspnea were not included if their condition had not improved within this time. Standard clinical and biological data were also collected. Blood analyses were performed at the discretion of the physician in charge of the patient. A follow-up visit conducted by a chest physician between 30 and 45 days after inclusion (stable phase) included pulmonary function tests and the same questionnaires as during exacerbation. If the participant showed persistent signs of exacerbation during the follow-up visit, the visit was postponed for a further 15 days. If, at this second follow-up, the participant was still not considered to have returned to a stable clinical state, he/she was excluded from the analysis.

### Measurements and outcomes

Pain was assessed using two self-reported scales, the short-form McGill Pain Questionnaire (SF-MPQ, validated for the rating of the intensity and quality of pain) [15] (primary outcome) and the short form of the Brief Pain Inventory (BPI, a pain assessment tool that assesses intensity and the effect of pain on function) [16] (secondary outcome). Secondary study outcomes were to investigate the differences in pain locations (as reported by patients using the diagram proposed in the BPI) between the exacerbation and stable phases and to investigate correlations between (1) pain intensity (SF-MPQ) and dyspnea intensity (Borg Dyspnoea Scale [17]) and (2) pain intensity (SF-MPQ) and symptoms of anxiety or depression (Hospital Anxiety and Depression Scale (HADS) [18]). Differences in pain intensity (SF-MPQ) according the GOLD stage and the exacerbation severity using the BAP-65 score (elevated Blood Urea Nitrogen, Altered mental status, Pulse > 109 beats/min, age > 65 years [19]) or the need for noninvasive or invasive ventilation or intensive care unit admission were also compared.

At inclusion, the following variables were also collected to describe participant characteristics; previous AECOPD and GOLD stage; comorbidities and treatments; details of any analgesic treatment in the previous 24 hours; vital parameters at admission; blood analysis; as well as final diagnosis and hospital outcomes. These data were retrieved from medical chart reports of previous consultations, and if the medical history was unclear or not documented, participants and/or general practitioners were asked for clarification. During the stable phase follow-up visit, information about analgesia and COPD treatments was again recorded. During this follow-up visit, spirometry was performed, including pre and post-bronchodilation measurements of forced expiratory volume in one second (FEV1) and forced vital capacity (FVC). The FEV1/FVC ratio was then calculated.

### Statistical analysis

The sample size calculation showed that a sample of 50 patients was necessary to achieve > 90% power to detect a mean paired clinical difference of 5 points on the SF-MPQ with an estimated standard deviation of differences of 10 and a significance level (alpha) of 0.05 using a two-sided Wilcoxon test and assuming normal distribution (PASS 15, NCSS, LLC. Kaysville, Utah, USA). The minimal clinically important difference for the SF-MPQ has been found to be 5 points in various populations [15]. The estimated standard deviation of differences was estimated from stable patient populations because, to our knowledge, no studies have reported SF-MPQ data during the exacerbation phase [5,6]. Quantitative parameters are reported as medians and interquartile ranges (IQR). Categorical and qualitative variables are reported as numbers and percentages. Wilcoxon rank sum tests were used to analyze the differences in pain intensity measured by the SF-MPQ and the BPI between the exacerbation and stable phases (primary outcome). Kruskal-Wallis and Chi2 tests were used to compare the intensity of pain on the SF-MPQ in different subgroups according to the severity of the COPD and the severity of the exacerbation. Finally, a Pearson test was used to evaluate the correlation between other quantitative parameters (dyspnea, anxiety and depression scores) and the SF-MPQ. Missing data were not replaced. Statistical analysis was conducted using SPSS software (v20, IBM, NY, USA). Dataset is available in S1 Table and variable description is available in S1 Dictionary.

## Results

**Fig 1:**
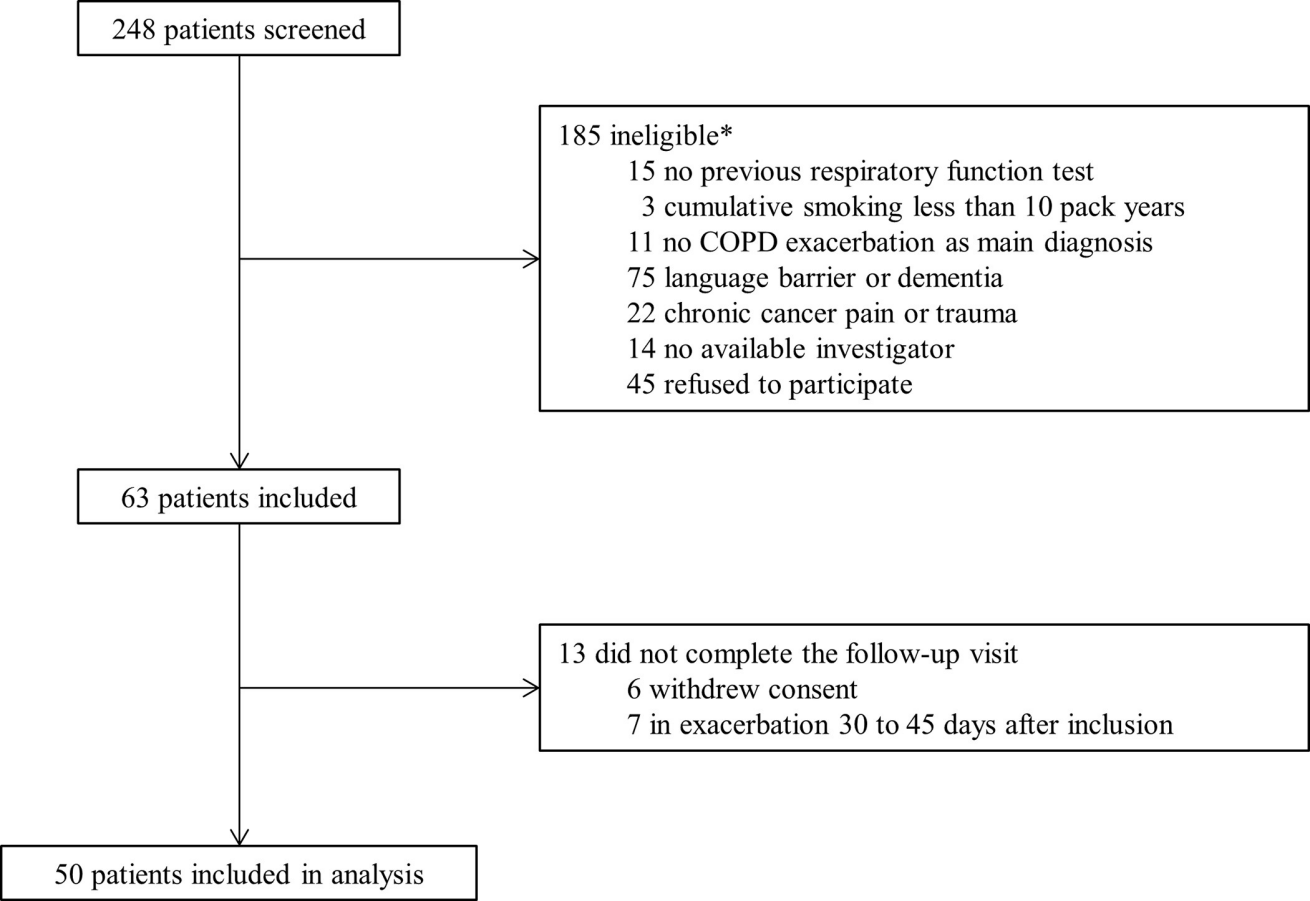
Study profile. *Patients could have more than one exclusion criterion.

Between April 2016, and January 2018, 248 patients admitted for an AECOPD were screened and 50 were included. The study profile is shown in Fig 1 and the characteristics of the participants at inclusion are presented in Tables 1 and 2. The median delay between admission and the follow-up visit was 36 days [31–45].

**Table 1:** Participant characteristics.

Characteristics	n = 50
Sex	
Male (n (%))	33 (66)
Age (years)	69 [61–78]
Body mass index (kg/m^2^)	22.9 [18.4–27.9]
FEV_1_ (% predicted)*	44 [33–48]
FVC (% predicted)*	68 [59–75]
FEV_1_ / FVC (%)*	46 [38–56]
**GOLD stage**	
I	2 (4)
II	19 (38)
III	21 (42)
IV	8 (16)
Current smoker (n (%))	16 (32)
Cumulative tobacco consumption (pack-years)	41 [31–58]
St. George’s Respiratory Questionnaire, total score (%)	71 [65–77]
Symptoms score (%)	61 [50–73]
Activity score (%)	87 [79–92]
Impact score (%)	64 [54–74]
**Comorbities**	
Asthma (n (%))	7 (14)
Hypertension (n (%))	20 (40)
Chronic cardiac failure (n (%))	5 (10)
Chronic renal failure (n (%))	3 (6)
**AECOPD in the previous year (n (%))**	
0	14 (28)
1	14 (28)
≥ 2	22 (44)
**Patients hospitalized during the previous year for AECOPD (n (%))**	
0	29 (58)
1	13 (26)
≥ 2	8 (16)
**Current medication (n (%))**	
Inhaled bronchodilators	50 (100)
Inhaled corticosteroids	25 (50)
Oral steroids (maintenance)	4 (8)
LTOT (n (%))	21 (42)
Home Non-invasive ventilation (n (%))	5 (10)
** Analgesic medication in the 24 hours prior to inclusion (n (%))**	
Non-opioid	18 (36)
Opioid	0 (0)

*: Spirometry was performed during the follow-up visit. Data are medians (IQR), or counts (%), unless otherwise stated. AECOPD: acute exacerbation of chronic obstructive pulmonary disease. FEV1: forced expiratory volume in 1 second. FVC = forced vital capacity. LTOT: long term oxygen therapy

**Table 2:** Characteristics of exacerbations.

Characteristics	n = 50
**Vital parameters at admission**	
Systolic arterial pressure (mmHg)	144 (129–153)
Heart rate (beats per minute)	101 (87–112)
SpO_2_ (%) in room air	93 (91–96)
Respiratory rate (per minute)	24 (20–34)
Body temperature (°C)	37.1 (36.7–37.6)
**Blood gases in room air**	
PaO_2_ (kPa)	9.05 (7.71–10.53)
PaCO_2_ (kPa)	5.44 (4.56–6.43)
pH	7.42 (7.38–7.46)
Bicarbonates (mmol/L)	25.3 (23.6–29.0)
BAP-65	2 [1–3]
**Treatment in the emergency department (n (%))**	
Oxygen	34 (68)
Noninvasive ventilation	10 (20)
Invasive ventilation	0 (0)
Short acting bronchodilators	46 (92)
Oral steroids	29 (58)
Antibiotics	22 (44)
**Hospital outcomes (n (%))**	
Discharged from the emergency department (n (%))	13 (26)
Hospitalization in medical ward (n (%))	35 (70)
Length of hospitalization (days)	5 (3–8)
Hospitalization in intensive care units (n (%))	2 (4)
Length of stay in intensive care unit (days)	5 (5–5)
Death (n (%))	0 (0)

Data are medians (IQR), or counts (%), unless otherwise stated. BAP-65 score: Blood Urea Nitrogen test (BUN > 25 or Urea > 9), Altered mental status, Pulse > 109 bpm and Age > 65 score. Each item in the BAP-65 counts for 1 (0 to 4), a higher score indicates a more severe prognosis in acute exacerbation of chronic obstructive pulmonary disease.

### Pain intensity

**Fig 2:**
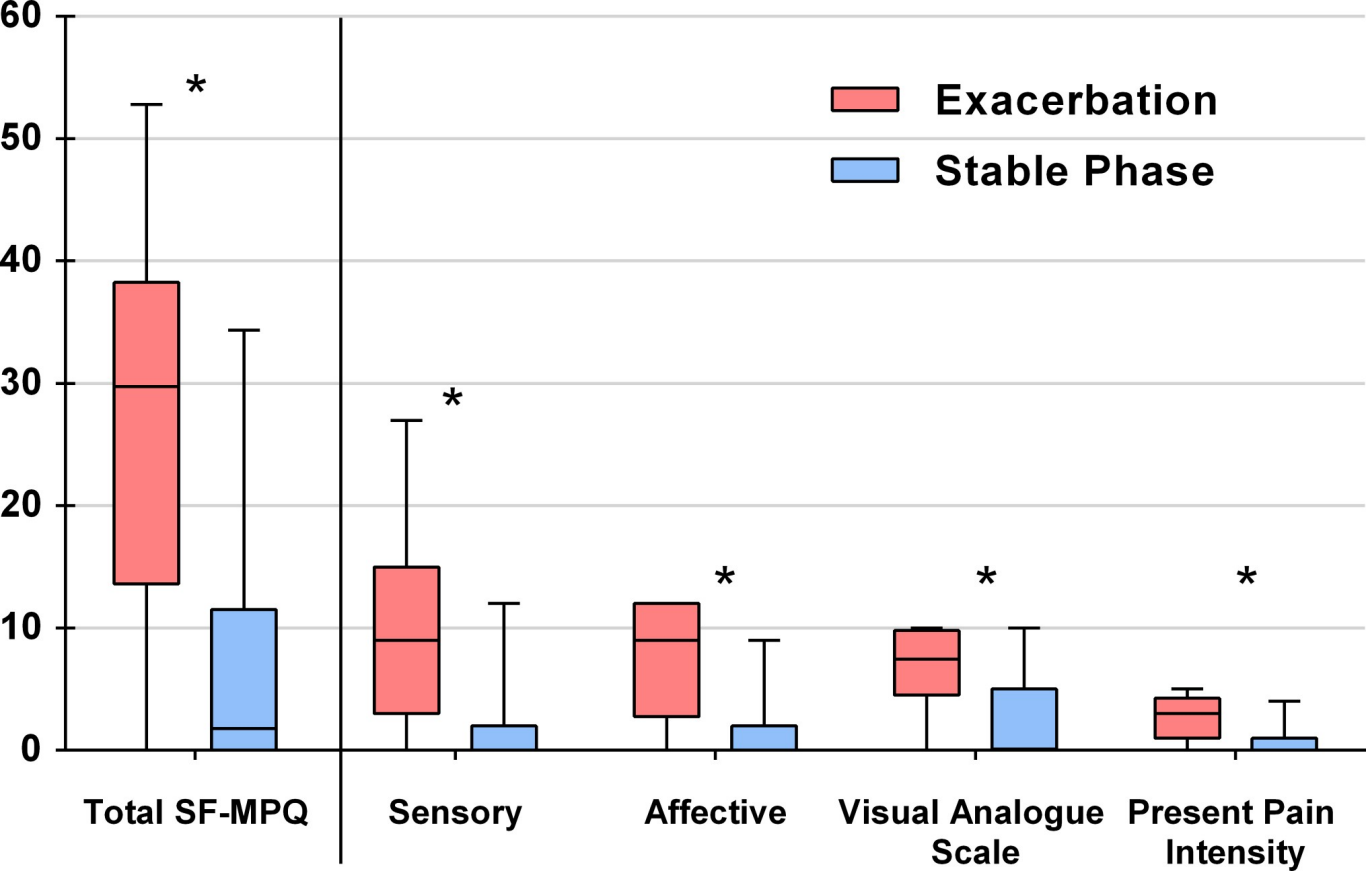
Short Form McGill Pain Questionnaire (SF-MPQ) and its components during the exacerbation and stable phases. * p-values are <0.001 for all comparisons between the exacerbation and stable phases. Results are displayed on box-and-whisker plots, showing the intensity of reported pain levels with medians, interquartile ranges and ranges.

According to the SF-MPQ results, 46 (92%) patients reported pain during the AECOPD compared to 29 (58%) in the stable phase (p<0.001). The results of the BPI indicated that one (2%) patient had no pain during exacerbation and that six (12%) had no pain during the stable phase. All patients (except one) who reported no pain on the BPI also reported no pain on the SF-MPQ (one patient had a SF-MPQ = 1 and a BPI = 0). Reported pain intensity evaluated by the SF-MPQ was significantly higher during exacerbation than the stable phase (29.7 [13.6–38.2] vs. 1.4 [0.0–11.2]; p<0.001) with significant differences for all sub-parts of the SF-MPQ (Fig 2). During exacerbation, 15 (30%) patients received analgesia in the emergency department: 14 patients received only paracetamol while one patient received paracetamol, ketoprofene and tramadol. Non-pharmacological pain treatments were not used during exacerbation although some patients may have benefited from self-hypnosis as several health-professionals were trained in this technique. During the stable phase, 19 (38%) patients reported taking analgesia: 17 patients took only paracetamol and two patients took paracetamol and tramadol.

**Fig 3:**
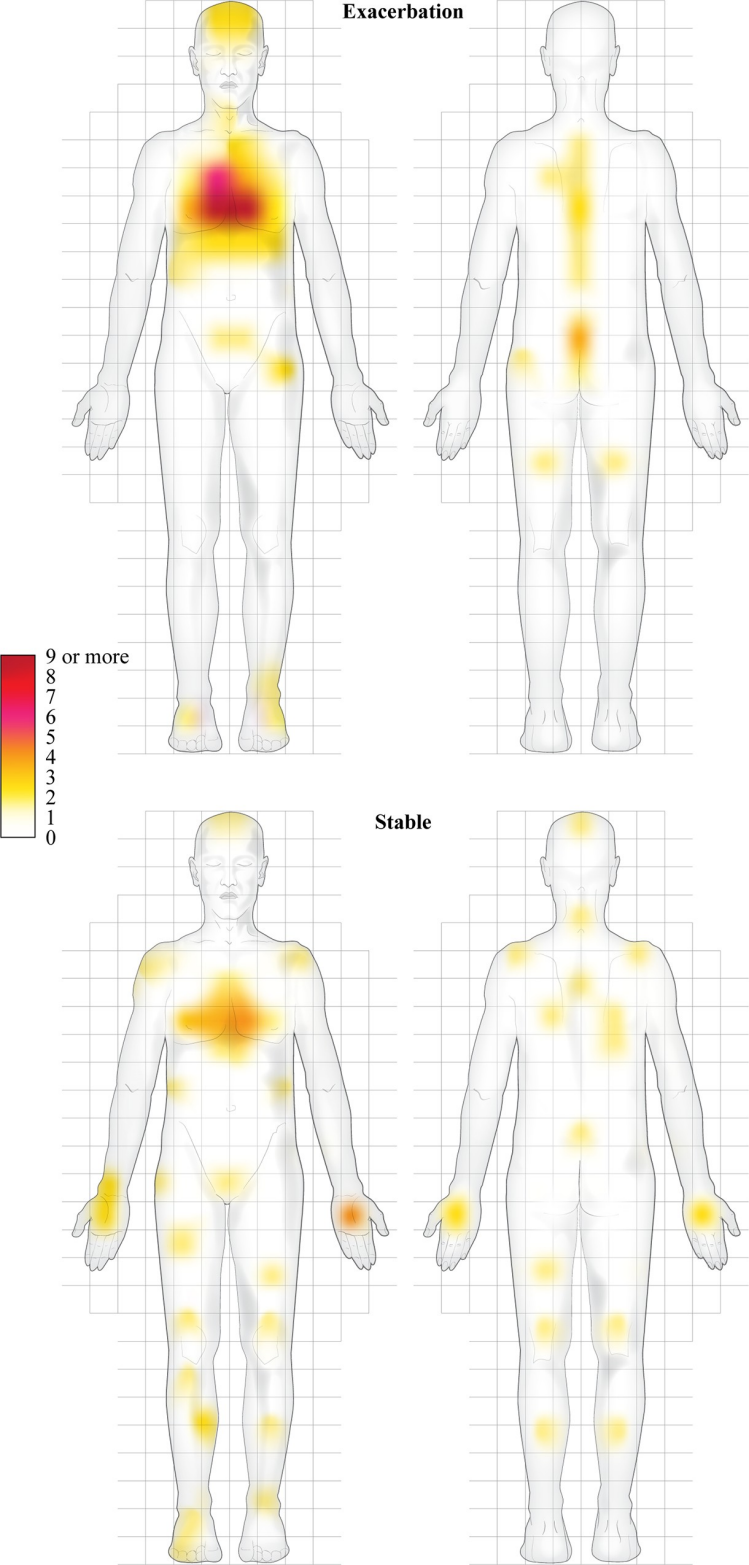
Location of pain during the exacerbation and stable phases. The grid was used to count each mark made by a patient. Patients could mark one or more areas.

Pain intensity measured by the BPI was also higher during exacerbation (exacerbation: 61 [37–79], stable phase: 25 [9–47]; p<0.001). According to the BPI diagram, during exacerbation, 38 patients reported pain predominantly located in the chest, whereas only three reported pain in their arm. During the stable phase, of the 44 patients who reported pain, only 13 reported chest pain and 10 reported arm pain (p = 0.02, Fig 3). The highest pain intensity was 7 [6–9] during exacerbation and 3 [0 – 5] during the stable phase (p<0.001). Mean pain intensity was 3 [2–5] during exacerbation and 3 [0 – 5] during the stable phase (p = 0.19).

### Dyspnea, anxiety and depression

Borg Dyspnea Scale scores were higher during the exacerbation (7 [5–8]) than the stable phase (5 [3–7]) (p<0.001). There was no difference in intensity of either anxiety (12 [8–17] vs. 11 [8–14], p = 0.13) or depression symptoms (13 [7–15] vs. 10 [7–15], p = 0.08 between the exacerbation and stable phases.

### Pain intensity and other variables

Pain intensity (SF-MPQ) was significantly correlated with Borg scale scores (Pearson’s r = 0.37, p = 0.008), anxiety (Pearson’s r = 0.60, p<0.001) and depression symptoms (HADS) (Pearson’s r = 0.42, p = 0.002) during exacerbation. During the stable phase, pain intensity was only correlated with Borg scale scores (Pearson’s r = 0.37, p = 0.007). In multiple regression analysis, only correlation between pain intensity (SFMPQ) and anxiety remained significant during exacerbation (p<0.001), and between pain intensity (SFMPQ) and Borg scale scores at stable phase (p = 0.01). In univariate analysis showed pain intensity tended to be higher in patients with stage IV COPD both during exacerbation (p = 0.06) and in the stable phase (p = 0.15). Pain intensity was higher in patients with lower BAP-65 scores (score ≤ 1: 30.9 [17.7–38.2] vs. score ≥ 2: 19.3 [3.2–36.6]; p = 0.046) but did not differ between patients who required standard care and those who received ventilation or intensive care (standard: 30.9 [15.8–38.2] vs. ventilation or intensive care: 23.0 [15.3–33.2]; p = 0.88].

## Discussion

This study is the first to report high levels and prevalence of pain in AECOPD. Almost all participants reported pain during exacerbation and just over half during the stable phase. Pain intensity was also much higher during exacerbation compared with the stable phase. Pain was predominantly located in the chest during exacerbation and pain location shifted in the stable phase.

The pain intensity during exacerbation found in the present study was higher than reports of pain intensity in other diseases. For example, mean cumulated sensory and affective SF-MPQ rating in patients with malignant pleural mesothelioma [20], musculoskeletal pain [15] or burns [21] has been found to be respectively 15.0, 16.9 and 15.1, all of which are much lower than the maximum reported value of 52.8 during exacerbation in our cohort, suggesting that AECOPD is associated with considerable levels of pain. Although the median value for the cumulated components of the SF-MPQ was high during exacerbation, much of this can be attributed to the affective component and explains why the SF-MPQ and anxiety scores correlated during exacerbation but not in the stable phase. Few studies have evaluated pain associated with respiratory problems, therefore it is difficult to compare our results. One study showed that 79% of patients with cystic fibrosis in acute exacerbation experienced pain [22]. Mean pain intensity and the highest pain intensity (BPI components) during exacerbation were both rated as 3 during in that study. In the present study of individuals with COPD, the highest pain intensity was greater (7 [6–9]) while mean pain was similar (3 [2–5]). This could indicate that pain experienced during AECOPD might be mostly due to paroxysms of breathing.

Pain was correlated with dyspnea intensity during the stable phase. This could be explained by similar perception-mechanisms for pain and dyspnea and the sharing of some cortical areas for processing [12]. Clinical assessments during AECOPD usually include the measurement of both dyspnea and anxiety, but the results of the present study suggest that pain is also an important characteristic of AECOPD and so should be included in these routine evaluations. It is interesting to note that only 30% of participants received analgesia during the exacerbation phase and 38% during the phase stable; contrasting considerably with the rate of participants who reported pain (92% during the exacerbation phase and 58% during the stable phase). Furthermore, in most cases, only paracetamol was administered, despite the high intensity of pain reported. Paracetamol could also have been administered as an antipyretic treatment in the context of AECOPD. These data suggest that current pain treatment is insufficient in patients with COPD, especially during exacerbations. Pain is known to influence treatment compliance including rehabilitation [13,14], therefore it seems reasonable to suggest that increased levels of analgesia are warranted to reduce pain during AECOPD, perhaps even in patients who require noninvasive ventilation [23]. The choice of treatment is complex due to the different levels of pain severity in each patient, as well as the emotional component of pain. Treatments, whether pharmacological or not [24], should provide potent analgesia as well as reduce anxiety, while of course remaining safe. Benzodiazepines and high-doses opioids (>30mg morphine equivalent a day) have been shown to be associated with poor outcomes in patients with COPD, at least during the stable phase [25,26].

One surprising finding from our results was the change in the location of pain between the exacerbation phase where pain was almost exclusively reported in the chest, to the stable phase where pain was reported equally in the chest and arms. Previous studies have reported that patients with stable COPD mainly have pain in their upper arms, shoulders and neck [7,27]. In the current study, however, participants reported more distally, in the limbs. Chest pain has been reported by 30% to 50% of patients with stable COPD [11], a similar percentage to the numbers in the present study. In the present study, pain during AECOPD was mainly located in the chest, back and head and it would seem reasonable to attribute this to the respiratory muscle overload, hypercapnia and the bronchial and systemic inflammation that occur during exacerbations. We believe that changes in the location and intensity of pain that occurred between the stable and exacerbation phases should be investigated for the early prediction of an exacerbation. The intense pain described by patients could also represent an important, but currently underestimated, contribution to the changes in daily physical activity in patients with COPD [28].

This study has several limitations. Firstly, only patients with mild exacerbations of COPD, as shown by their blood gas values, vital signs and treatments were included. Patients who had severe exacerbations were unable to reliably answer the questionnaires due to decreased consciousness or major dyspnea and were not included. The main reasons for not including patients were refusal to participate, cognitive impairment or the presence of chronic, cancer-related pain. We believe that the relatively low inclusion rate is representative of the proportion of elderly, comorbid and anxious individuals with AECOPD who present to the emergency department. Another possible barrier to inclusion may have been the prospect of a mandatory 1-month visit for pulmonary function tests, which may have put off individuals in exacerbation and presenting at the emergency department from participating. Secondly, we did not use a specific pain scale to assess neuropathic pain although peripheral neuropathy may be present in patients with COPD [29]. Nonetheless, the SF-MPQ has been validated for use in neuropathic and non-neuropathic pain [15]. We also did not extensively documented patient comorbidities. Some comorbidities such as arthritis or osteoporosis could impact pain evaluation. However, the pain reports and ratings during the stable phase of COPD were similar to those previously described for COPD [5,7]. This suggests that the changes found during the exacerbation phase, and thus the transition from a stable to an exacerbated COPD state, are reliable. However, Borg dyspnea scores were significantly reduced but not completely normalized, which could indicate that the exacerbation was not completely resolved. Seven participants were excluded during the follow-up because they reported not having completely recovered from the initial exacerbation. All the other participants were considered to be in a stable phase. Third, the 30–45 day delay is in accordance with the 4–6 week first follow-up visit recommended by the 2013 GOLD guidelines [2].Fourth, the study was likely underpowered for the analysis of secondary factors such as the intensity of anxiety and depression symptoms, or pain intensity between participants who required standard care and those who received ventilation or intensive care, which might explain the lack of differences. The results of these secondary analyses must therefore be considered as exploratory. Finally it is possible that reporting bias may have lowered pain ratings in the stable phase, since participants might have remembered their previous answers during the exacerbation. However, it should also be remembered that the two evaluations were over one calendar month apart. Therefore, the actual effect of this recall is likely to have been only minimal [30]. As a result of the conclusions drawn above, we believe the results of this study are robust, although it should be noted for future work that this study was not sufficiently powered to detect a correlation between pain intensity and COPD severity or exacerbation severity.

## Conclusions

This study found a higher prevalence and intensity of pain and a difference in pain locations during COPD exacerbations compared to the stable phase. During exacerbation, pain ratings were correlated with both dyspnea and anxiety. Based on the results of the present study, we suggest the assessment and treatment of pain should be integrated into multimodal care approaches for AECOPD in order to improve patient centered outcomes. New studies are also required to evaluate pain management during AECOPD and the consequences of pain on patient outcomes.

## Supporting information

S1 Dictionary Description of the variables. (CSV)

S1 Table Complete dataset to replicate the analyses. The dataset includes: participant characteristics and history; exacerbations characteristics; questionnaire results at inclusion and during the stable phase.
(CSV)

## References

[1] Tang CY, Taylor NF, McDonald CF, Blackstock FC. Level of adherence to the GOLD strategy document for management of patients admitted to hospital with an acute exacerbation of COPD. Respirology 2014; 19(8):1191–7. 10.1111/resp.12361 25123950

[2] GOLD—the Global initiative for chronic Obstructive Lung Disease. https://goldcopd.org/wp-content/uploads/2018/11/GOLD-2019-v1.7-FINAL-14Nov2018-WMS.pdf. Accessed/ 18 February 2019.

[3] Lopez-Campos JL, Bustamante V, Muñoz X, Barreiro E. Moving towards patient-centered medicine for COPD management: multidimensional approaches versus phenotype-based medicine—a critical view. COPD 2014;11(5):591–602. 10.3109/15412555.2014.898035 24914771

[4] Andenæs R, Momyr A, Brekke I. Reporting of pain by people with chronic obstructive pulmonary disease (COPD): comparative results from the HUNT3 population-based survey. BMC Public Health. 2018 1 25;18(1):181 10.1186/s12889-018-5094-5 29370850 PMC5785865

[5] HajGhanbari B, Holsti L, Road JD, Darlene Reid W. Pain in people with chronic obstructive pulmonary disease (COPD). Respir Med 2012;106(7):998–1005. 10.1016/j.rmed.2012.03.004 22531146

[6] HajGhanbari B, Garland SJ, Road JD, Reid WD. Pain and physical performance in people with COPD. Respir Med 2013;107(11):1692–9. 10.1016/j.rmed.2013.06.010 23845881

[7] van Dam van Isselt EF, Groenewegen-Sipkema KH, Spruit-van Eijk M, Chavannes NH, de Waal, Janssen DJA, et al Pain in patients with COPD: a systematic review and meta-analysis. BMJ Open 2014;4(9):e005898 10.1136/bmjopen-2014-005898 25260370 PMC4179414

[8] Bentsen SB, Rustøen T, Miaskowski C. Prevalence and characteristics of pain in patients with chronic obstructive pulmonary disease compared to the Norwegian general population. J Pain 2011;12(5):53945. 10.1016/j.jpain.2010.10.014 21549316

[9] Borge CR, Wahl AK, Moum T. Pain and quality of life with chronic obstructive pulmonary disease. Heart Lung 2011;40(3):e90–101. 10.1016/j.hrtlng.2010.10.009 21444112

[10] Lee AL, Harrison SL, Goldstein RS, Brooks D. An exploration of pain experiences and their meaning in people with chronic obstructive pulmonary disease. Physiother Theory Pract 2018;34(10):765–72. 10.1080/09593985.2018.1425512 29319390

[11] Janssen DJA, Wouters EFM, Parra YL, Stakenborg K, Franssen FME. Prevalence of thoracic pain in patients with chronic obstructive pulmonary disease and relationship with patient characteristics: a cross-sectional observational study. BMC Pulm Med 2016;16:47 10.1186/s12890-016-0210-8 27052199 PMC4823883

[12] Schön D, Rosenkranz M, Regelsberger J, Dahme B, Büchel C, von Leupoldt A. Reduced perception of dyspnea and pain after right insular cortex lesions. Am J Respir Crit Care Med 2008;178(11):1173–9. 10.1164/rccm.200805-731OC 18776150

[13] Harrison SL, Lee AL, Elliott-Button HL, Shea R, Goldstein RS, Brooks D, et al The role of pain in pulmonary rehabilitation: a qualitative study. Int J Chron Obstruct Pulmon Dis 2017;12:3289–99. 10.2147/COPD.S145442 29184398 PMC5685149

[14] Chen Y-W, Camp PG, Coxson HO, Road JD, Guenette JA, Hunt MA, et al A Comparison of Pain, Fatigue, Dyspnea and their Impact on Quality of Life in Pulmonary Rehabilitation Participants with Chronic Obstructive Pulmonary Disease. COPD 2018;15(1):65–72. 10.1080/15412555.2017.1401990 29227712

[15] Strand LI, Ljunggren AE, Bogen B, Ask T, Johnsen TB. The Short-Form McGill Pain Questionnaire as an outcome measure: test-retest reliability and responsiveness to change. Eur J Pain 2008;12(7):917–25. 10.1016/j.ejpain.2007.12.013 18289893

[16] Tan G, Jensen MP, Thornby JI, Shanti BF. Validation of the Brief Pain Inventory for chronic nonmalignant pain. J Pain 2004;5(2):133–7. 10.1016/j.jpain.2003.12.005 15042521

[17] Johnson MJ, Close L, Gillon SC, Molassiotis A, Lee PH, Farquhar MC, et al Use of the modified Borg scale and numerical rating scale to measure chronic breathlessness: a pooled data analysis. Eur Respir J 2016;47(6):1861–4. 10.1183/13993003.02089-2015 26989107

[18] Bratås O, Grønning K, Forbord T. Psychometric properties of the Hospital Anxiety and Depression Scale and The General Health Questionnaire-20 in COPD inpatients. Scand J Caring Sci 2014;28(2):413–20. 10.1111/scs.12054 23713548

[19] Tabak YP, Sun X, Johannes RS, Gupta V, Shorr AF. Mortality and need for mechanical ventilation in acute exacerbations of chronic obstructive pulmonary disease: development and validation of a simple risk score. Arch Intern Med 2009;169(17):1595–602. 10.1001/archinternmed.2009.270 19786679

[20] MacLeod N, Kelly C, Stobo J, McMahon L, Taggart D, Fallon M, et al Pain in Malignant Pleural Mesothelioma: A Prospective Characterization Study. Pain Med 2016;17(11):2119–26. 10.1093/pm/pnw061 27117437

[21] Mason ST, Arceneaux LL, Abouhassan W, Lauterbach D, Seebach C, Fauerbach JA. Confirmatory Factor Analysis of the Short Form McGill Pain Questionnaire With Burn Patients. Eplasty 2008;8:e54 19119306 PMC2596340

[22] Kelemen L, Lee AL, Button BM, Presnell S, Wilson JW, Holland AE. Pain impacts on quality of life and interferes with treatment in adults with cystic fibrosis. Physiother Res Int. 2012;17(3):132–41. 10.1002/pri.524 22025449

[23] Longrois D, Conti G, Mantz J, Faltlhauser A, Aantaa R, Tonner P. Sedation in non-invasive ventilation: do we know what to do (and why)? Multidiscip Respir Med 2014;9(1):56 10.1186/2049-6958-9-56 25699177 PMC4333891

[24] Mulhall P, Criner G. Non-pharmacological treatments for COPD. Respirology 2016;21(5):791–809. 10.1111/resp.12782 27099216

[25] Ekström MP, Bornefalk-Hermansson A, Abernethy AP, Currow DC. Safety of benzodiazepines and opioids in very severe respiratory disease: national prospective study. BMJ 2014;348:g445 10.1136/bmj.g445 24482539 PMC3906915

[26] Vozoris NT, Wang X, Fischer HD, Bell CM, O’Donnell DE, Austin PC, et al Incident opioid drug use and adverse respiratory outcomes among older adults with COPD. Eur Respir J 2016;48(3):683–93. 10.1183/13993003.01967-2015 27418553

[27] Lohne V, Heer HCD, Andersen M, Miaskowski C, Kongerud J, Rustøen T. Qualitative study of pain of patients with chronic obstructive pulmonary disease. Heart Lung 2010;39(3):226–34. 10.1016/j.hrtlng.2009.08.002 20457343

[28] Esteban C, Quintana JM, Garcia-Gutierrez S, Anton-Ladislao A, Gonzalez N, Baré M, et al Determinants of change in physical activity during moderate-to-severe COPD exacerbation. Int J Chron Obstruct Pulmon Dis 2016;11:251–61. 10.2147/COPD.S79580 26893555 PMC4745854

[29] Aras YG, Aydemir Y, Güngen BD, Güngen AC. Evaluation of central and peripheral neuropathy in patients with chronic obstructive pulmonary disease. Int J Chron Obstruct Pulmon Dis 2018;13:1857–62. 10.2147/COPD.S159738 29922052 PMC5995289

[30] Daoust R, Sirois M-J, Lee JS, Perry JJ, Griffith LE, Worster A, et al Painful Memories: Reliability of Pain Intensity Recall at 3 Months in Senior Patients. Pain Res Manag 2017;2017:5983721 10.1155/2017/5983721 28260963 PMC5312450

